# Investigating falls in adults with intellectual disability living in community settings and their experiences of post-fall care services: protocol for a prospective observational cohort study

**DOI:** 10.1186/s12877-018-0862-8

**Published:** 2018-07-30

**Authors:** Portia Ho, Caroline Bulsara, Shane Patman, Max Bulsara, Jenny Downs, Anne-Marie Hill

**Affiliations:** 1 School of Physiotherapy, The University of Notre Dame Australia, 19 Mouat St, PO Box 1225, Fremantle, WA 6959 Australia; 2 Institute for Health Research, The University of Notre Dame Australia, 19 Mouat St, PO Box 1225, Fremantle, WA 6959 Australia; 3 Telethon Kids Institute, 100 Roberts Road, Subiaco, PO Box 855, West Perth, Western 6872 Australia; 4 School of Physiotherapy and Exercise Science, Curtin University, GPO Box U1987, Perth, WA 6845 Australia

**Keywords:** Intellectual disability, Accidental falls, Falls rate, Barriers, Mixed methods

## Abstract

**Background:**

Falls among older adults with intellectual disability (ID) are recognised as a serious health problem potentially resulting in reduced health-related quality of life and premature placement in residential care. However there are limited studies that have investigated this problem and thus falls rates among older adults with ID remain uncertain. Furthermore, people with ID rely heavily on familial and professional care support to address health problems, such as after having a fall. No studies have explored the post-fall care that people with ID receive.

**Method:**

This research will be carried out in two phases using a convergent mixed methods design. The aim of Phase 1 is to estimate the falls rate by prospectively observing a cohort of older adults (≥ 35 years) with ID (*n* = 90) for six months. Phase 1 will be conducted according to STROBE guidelines. In Phase 2, participants from Phase 1 who have experienced a fall(s) will be asked to participate in a semi-structured interview to explore their post-fall experience.

**Discussion:**

This study will determine the rate of falls among older adults with ID living in community based settings, which will assist to identify the extent of this problem. Data collected from the study will also aid in understanding the circumstance of falls and related falls risk factors in this cohort. This will include exploring any barriers that older adults with ID may encounter when seeking or undertaking recommended post-fall care advice. Findings from this research will potentially inform future development of falls prevention services for older adults with ID. This study has been approved by the University Human Research Ethics Committee.

**Trial registration:**

The protocol for this study is registered with the Australian New Zealand Clinical Trial Registry (ACTRN12615000926538) on 7 September 2015. www.anzctr.org.au/Trial/Registration/TrialReview.aspx?id=368990&isReview=true

## Background

Falls are a serious health problem which result in substantial socio-economic costs, with estimates that one third of people aged 65 years and over, who are living in the community, experience at least one fall per year [1, 2]. Falls are the leading cause of injury amongst older Australians [3]. In 2011–12 there were nearly 96,000 hospitalisations from falls related injuries in Australia, with each hospital stay lasting an average of eight days [3]. The healthcare cost associated with these falls was estimated to be $498.2 million in 2009 and this is expected to increase to approximately $1.4 billion by 2051 [4].

Falls among older adults with intellectual disability (ID) living in the community are also a cause for concern. People with ID have been observed to experience signs of ageing from their third decade in life [5, 6], and as such are considered ‘older adults.’ At least 50% or more of these falls result in injury [7, 8] and between 6 and 11% result in severe injuries, such as fractures and concussion [8, 9]. Qualitative research undertaken among adults with ID has found that falls may lead to reduced activity and independence and increase the burden of care on their caregivers [10]. Through observation, it has been suggested that people with ID have increased risk of institutionalisation from having falls [11]. Few studies have investigated falls rates amongst people with ID living in community based settings. Estimates range from 0.93 to 6.29 falls per person year [8, 9, 12, 13]. Other studies have reported between 25 and 40.1% of people with ID experienced a fall(s) during the observation period, but did not report the number of falls [7, 14–16]. Only three studies provided sufficient data to estimate injurious falls rates and these were 0.38–0.73 injurious falls per person year [8, 9, 13]. Research guidelines recommend reporting falls and injurious falls rates, as well as the proportion of people who fall when conducting falls related studies [16]. Additionally only one study implemented prospective daily recording of falls, [8] which is recommended for falls prevention trials [16] to ensure accurate and comprehensive collection of falls data. Limitations to these study designs, poses uncertainty about the extent of the problem of falls among older adults with ID. Additionally a limited amount of studies have investigated risk factors and preventive strategies for falls among people with ID [7, 12, 15]. A review which examined risk factors and preventative strategies for falls prevention in people with ID was only able to include seven studies, and found there was a lack of evidence about falls management for people with ID, prompting authors to recommend that more research in this area be undertaken [17].

It can be challenging to conduct robust research with older adults with ID [18] and conducting falls research with this group is no exception. There are ethical and legal considerations in gaining informed consent [19] and extensive adaptations are recommended to maximise the participation of people with ID [20]. Falls recommendations for older people have been developed from established evidence from large meta-analyses in the older community dwelling population [21], however these recommendations are not specifically applicable to people with ID [22]. The perspectives that older adults with ID have about falls and falls prevention services are also unknown, as there have been no studies which have sought to understand what experiences older adults with ID have when they fall and subsequently seek access to existing falls prevention services.

The primary aim of the study is to investigate the rate of falls in older adults with ID living in community based settings. The secondary aims of the study are to i) investigate the rate of injurious falls, ii) determine the risk factors for falls in this population, iii) explore the participant’s experience when seeking health services after having a fall, and iv) investigate barriers that participants may encounter when seeking to undertake evidence based falls prevention strategies that are recommended by the health service.

## Methods

### Design

**Fig. 1:**
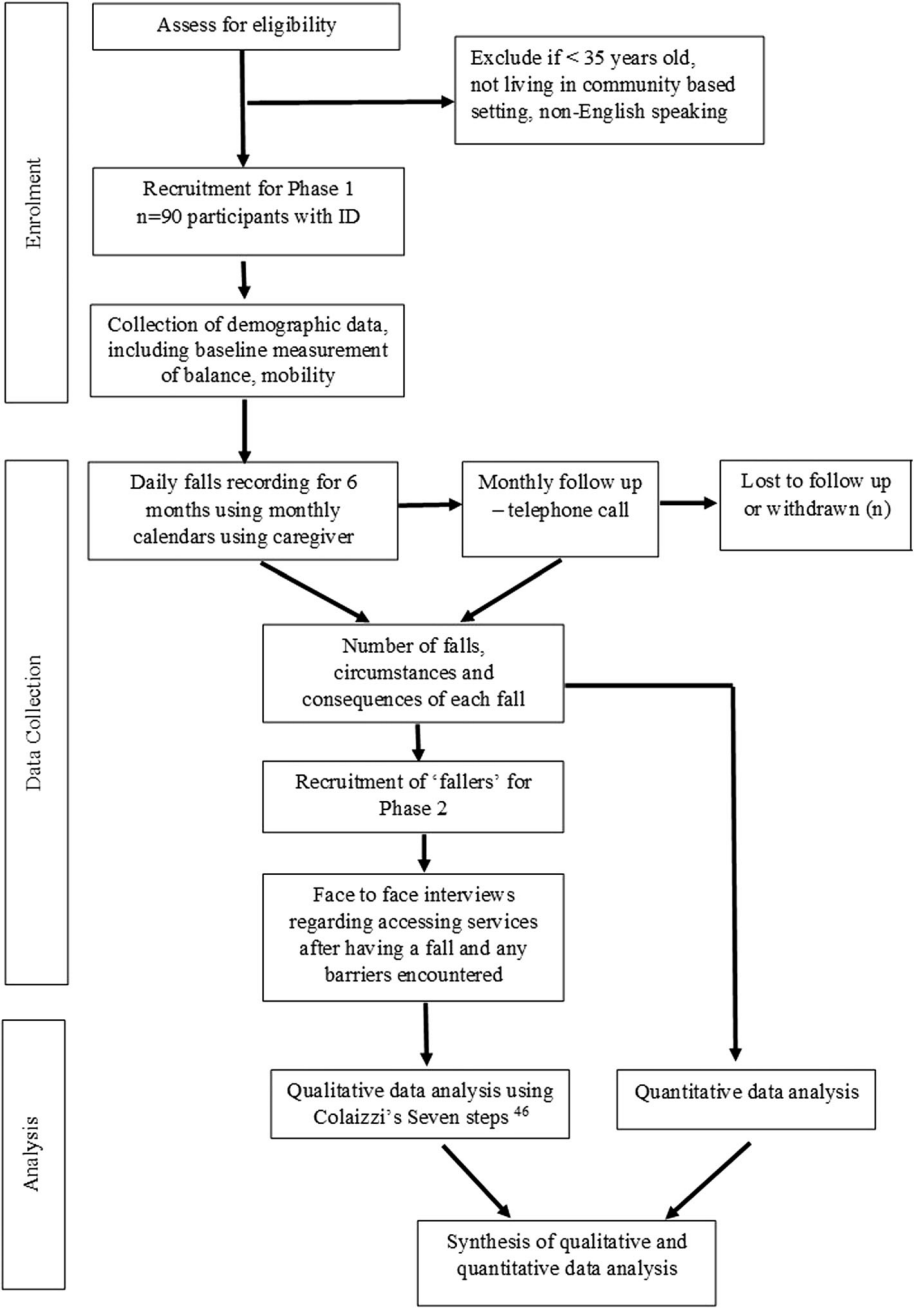
Participant flow through the study

A convergent mixed methods approach will be used in this study [23]. In Phase 1, a prospective observational cohort study will be conducted in accordance with STROBE guidelines [24]. In Phase 2, participants’ experiences of falls and access to falls prevention services will be explored using a descriptive phenomenological approach. Phenomenology is a research method used to describe a person’s lived experience of an event or ‘phenomenon’ [25]. In this case, it is the experience participants have when seeking healthcare services after a fall and the barriers that may be encountered while accessing or engaging in falls prevention recommendations. Data from the two phases will be analysed independently and the findings will subsequently be synthesised (Fig. 1).

### Ethics and informed consent

The study has received ethics approval from The University of Notre Dame Australia, Human Research Ethics Committee (015067F) and the affiliated local organisation for people with ID. All participants (or their legal representative or next-of-kin) will provide written informed consent to participate in the study. Three consent forms have been prepared: i) a consent form adapted to facilitate the participant’s understanding of the study and for what they are providing informed consent to, ii) a version for a family member or legal guardian to record their agreement for the person with ID to participate in the study and that in their opinion, the participant is not likely to object to participating in the study, and iii) a form for the caregiver(s) to provide informed consent that they are willing to support the participant with daily falls recordings and to facilitate communication with the researcher.

Ethical guidelines for research conducted in Western Australia (WA) contain information specific to adults who may lack the capacity to give consent [26]. These guidelines are also being followed when conducting this study. The guidelines specify that in accordance with the Guardianship and Administration Act [27], there is no provision for responsible persons to provide informed consent for a person who is assessed as being incapable of doing so, to take part in medical research [27]. Therefore in this study, where there is any uncertainty regarding the ability of the participant to provide consent, their guardian or next-of-kin will be asked to sign a separate consent form which records that they agree to the participant under their legal care participating in the study and that they believe the participant is not likely to object to participating in the study. Informed consent will be gained directly from the potential participant with ID where possible. The family member or caregiver will be asked to be present during this process to facilitate the participant’s understanding of what is involved in the study and witness the participant completing the consent form.

A step by step informed consent procedure will be carried out with each person with ID and their caregiver prior to determining if the person with ID is able to provide their own consent or if a proxy consent is required. The consent procedure consist of the researcher making several observations of the person with ID during the initial face to face meeting and during the discussion of the research. The researcher will ask the caregiver their opinion if the person with ID is able to make their own decisions. A short questionnaire is also administered to determine if the person with ID has understood the purpose of the study and the risk and benefits involved. The questionnaire will be administered in consideration of the communication style of the person with ID. The information gathered will allow the researcher to make a holistic informed decision regarding the person with ID’s ability to provide consent independently, or if a proxy consent is required.

After initial recruitment, the researcher maintains contact with the participant with monthly follow up, either by telephone or face to face, during the six month observational period. Telephone interviews are also conducted when the participant experiences a fall. These touch points provide an opportunity for the participant and the caregiver to raise any issues concerning data collection. It also provides the opportunity for the researcher to access the participant’s capacity to continue with the study and to seek verbal consent for continued participation. For participants providing consent independently, with their consent, the researcher will inform a suitable person who is involved in the participant’s care, to inform the researcher if there is a decline in cognitive function or health status. The researcher will follow with an assessment to determine their ability to provide consent and seek the legal representative for the participant where required, for continued participation.

In the later stages of the observation period, a caregiver who is involved in the care of a participant who experiences one or more falls may be asked to participate in an interview regarding their post-fall experience. Consent will be sought from the caregiver as well as the participant, for the caregiver to share their experiences with the researcher.

### Participants

People will be eligible for inclusion in the study if they have a confirmed diagnosis of ID or a diagnosis in which ID coexist, such as Down Syndrome, Retts Syndrome or Cerebral Palsy. People with ID in this study are defined by the Australian Psychological Society (APA), DSM – V criteria as people who present with limited intellectual and adaptive functioning [28]. This will be confirmed by their family or a caregiver. For the purpose of this project, adults with ID will be eligible if they are aged 35 years or older. Only older adults with ID living in the community will be eligible for inclusion. Community settings in Western Australia most often comprise of the older adult with ID living at home with their families, in independent units or in small group homes comprising of 2–9 housemates. Paid support caregivers are usually present for varying amounts of time within each living arrangement, to provide assistance with activities of daily living and community participation activities. Medical, allied health and social services are accessed through community options.

People with ID will be excluded if they are younger than 35 years old or living in a residential aged care facility such as a nursing home, or are in hospital. They will also be excluded from the study if they or their caregivers are unable to understand English.

### Setting and recruitment

Participants will be recruited from 2014 through to 2018, through a large non-for profit organisation that provides services to people with ID, operating in WA. This organisation provides an array of support services to over a thousand people with disability across the life span in the metropolitan and rural areas, including providing support with employment, accommodation, community participation, life-long learning and rehabilitation [29]. Approximately a quarter of the people for whom they provide services have a primary diagnosis of ID [30].

Participants will be recruited from advertisements and flyers posted on social media (Facebook and website) and within the common areas of the supporting organisation. Staff members of the organisation are also encouraged to provide study information to their eligible clients. Participants will be consecutively recruited until the target sample size of 90 participants is achieved. Participants who experience a fall in Phase 1 will be purposefully selected to participate in Phase 2 to ensure a diverse sample to capture data that will closely represent the experiences of older adults with ID when they seek healthcare services after experiencing a fall(s).

### Sample size

The incidence of falls in this population is uncertain, but based on previous studies is estimated to be about 0.27 [8, 12]. If the expected result is 0.27, we want 95% confidence, within a 1% absolute margin of error that the true incidence rate frequency lies between 0 and 1.27 [31]. Therefore a minimum sample size of 78 has been estimated to be required for Phase 1 of the study. As the study population has both physical and social disability and the observation period extends for six months, a drop out of approximately 15%, will be allowed for, which is higher than the 5% drop-out in a previous observational falls cohort study of older people [32]. Therefore the aim is to recruit 90 participants for Phase 1.

A purposeful sample [33] will be utilised for Phase 2. The aim is to purposefully select participants who have experienced a fall(s) in Phase 1 with different characteristics and/or consequences of the fall, in order to achieve a close representation of the experience older adults with ID have when accessing evidence based fall prevention recommendations. The aim is to be able to provide a holistic perspective about falls among people with ID and participants will be purposefully selected to represent those recruited. This sample will be guided by the participants who fall in Phase 1. Where possible, purposeful selection will include participants of different gender, age, diagnosis, level of independence and differing levels of communication (including verbal and non-verbal participants). Participants with different living arrangements (living with family or independently with or without support) and those who report varying circumstances surrounding their falls will also be considered when selecting the sample. Interviews will be continued until it is considered that saturation of themes has occurred [34] however it is understood that amongst this ‘hard-to-reach’ population this may not be entirely achievable.

### Outcome measures

The primary outcome of phase 1 will be the number of falls experienced by participants during the six month trial period. A fall is defined as ‘an event which results in a person coming to rest inadvertently on the ground or floor or other lower level’ [35]. Fall events will be collected prospectively with daily recordings of any fall occurrences for six months [14]. The participants will be provided with a monthly calendar for six months on which they, or their caregiver, will mark a cross on the day(s) if a fall occurs and draw a smiley face on day(s) where a fall is not experienced. The diary has a prominent phone number on it and participants are encouraged to contact the researcher at any time. It is envisaged that family and caregivers (paid and unpaid) will be providing substantial support and sometimes will be entirely relied upon to collect the falls data. If families or caregivers find that this method of data collection is not accurate, such as if daily recordings are not sustainable, other strategies may be discussed and put in place as appropriate. These strategies may include but not limited to, the researcher making contact more frequently, making contact with more than one caregiver or leaving a falls record form with questions from the post-fall telephone interview. Other strategies used are initiating contact with participants and also informing others who support the participant to be aware of their participation, to ensure that all falls are recorded. At the end of the month, the participants are asked to send the calendar back to the researcher. The researcher will follow up with a phone call at the end of every month to enquire if a fall has occurred or if they have trouble filling up the diary. When a fall occurs, participants are encouraged to contact the researcher. Caregivers are also encouraged to call when a fall is witnessed or when an unwitnessed fall is suspected. The details of the circumstances of the fall and any falls related injuries will be collected through a telephone call or face to face interview. This procedure follows recommended guidelines for conducting falls research [14]. Data about the fall such as, where and when the fall occurred, what happened at the time of the fall and if the fall is a result from a seizure will be collected. The number, type and location of any resulting injuries and any medical or first aid attention received will also be recorded. A fall will be classified as injurious if it results in bruising, laceration, dislocation, fracture or complaint of an onset of persistent pain as a result of the fall, which is concordant with previous research conducted in this area [32].

Baseline demographic data including age and other medical history such as diagnosis including number of comorbidities, any history of fracture, stroke or other condition affecting falls risk, number and type of medications, history of visual impairment and falls history, which have been previously identified as being risk factors for falls [9] will be collected. Other measures known to be associated with falls in people with ID [5, 13] that are collected at baseline will be body mass index, indoor and outdoor mobility, use and type of walking aid and classification of Intelligence Quotient score when known. Other supporting information about the participants that will be collected include education and employment history and level of care support. Functional measures of mobility using Timed-up and Go [36], balance using Four step-square test [37], and the Four-test balance Scale [38]. Five times Sit to Stand [39] will be used to measure lower limb strength. These physical outcome measures will be first carried out using standardised procedures [36–39]. Where required, the procedure will be modified appropriately and documented according to the participant’s communication styles and understanding. Strategies used to modify the instructions include the methods described in Salb et al.[40] which have demonstrated that the reliability of these tests used with people with ID is still maintained. Participants’ ability to perform activities of daily living will be measured using The Katz Index of Independence in Activities of Daily Living [41]. The Personal Well-being Index for people with ID [42] will be used to collect the participant’s perspective on their perceived health related quality of life (HRQoL). Where participants are unable to rate their HRQoL, the EQ-5D-5 L [43] will be used to ask caregivers to act as a proxy to rate the participant’s HRQoL as they perceive it.

Qualitative data gathered during Phase 2 of the study will explore the participants’ experience when accessing falls prevention services after experiencing a fall. These data will be collected using structured face-to-face interviews, which will be conducted and recorded by the researcher (PH), who is a therapist who is experienced in working with people with ID. Questions will be modified as appropriate using various strategies such as having a familiar family member and /or caregiver present to interpret the questions in the context in which the participant can understand. Participants who have limited verbal communication will be included in Phase 2. Where appropriate, alternative forms of communication (key word signs, augmentative and alternative communication devices, natural gestures) will be used to facilitate the interview. The researcher will provide additional support to the participant such as allowing time for them to express themselves, exploring the environment where the fall took place and where suitable drawing their attention to the part of their body where an injury was sustained.

Caregivers who were supporting the participant at the time of the fall or who provided support relating to treatment or intervention following the fall will be asked to participate in a separate interview, which will also be conducted by the researcher (PH). The participant (person with ID or a caregiver) will be asked to describe the events that took place after the fall with specific attention to any medical or healthcare services that they received. When required, the interviewer will prompt using a code, which has been designed around the recommended evidence based service pathway for falls prevention which is outlined in the WA falls model of care [44]. The interviewer will specifically ask the participant if they were seen by a medical provider after their fall, such as a medical hospital outpatient clinic or their family doctor for falls risk assessment, and if they were provided with any follow up referrals to an intervention such as physiotherapy for strength and balance exercises or an optometrist for a vision check. Open questions will be used to explore the experiences of the participants in further scope and depth. Participants will be asked if they sought and received the intervention or a plan was in place for them to undertake the intervention at a later date Depending on what the participants share, they will be asked if they encountered any barriers when seeking to undertake the intervention that was recommended.

### Statistical analysis

#### Quantitative data analysis

Data will be analysed using the SPSS 23.0 software package (SPSS Inc., Chicago, IL, USA) and STATA 14.0 (StataCorp. 2015. Stata Statistical Software: Release 14. College Station, TX: StataCorp LP). Descriptive statistics will be used to describe the clinical and demographic characteristics of the participants. Falls analyses will follow recommended statistical guidelines for analysing falls data [32, 45].

The primary outcome measure (rate of falls) will be expressed as an incidence rate per person year. The number of participants who experienced a fall will be expressed as a percentage of people falling in the cohort. The rate of injurious falls will be expressed as the incident rate per person year. Generalised linear mixed modelling will be conducted to examine participant and their clinical characteristics predictive of falls, injurious falls and the risk of falling in the six months after commencement of the falls diary.

The modelling strategies will start with unadjusted univariate analysis, followed by multivariate analysis, adjusting for age, severity of ID, presence or absence of epilepsy, level of assistance required with activities of daily living, level of mobility and a history of falls in the six months prior to enrolment in the study. These variables have been shown to be predictive of falls in either older people or people with ID.

#### Qualitative data analysis

Data collected from Phase 2 interviews will be analysed using Colaizzi’s seven stages of thematic analysis for phenomenological studies [25, 46]. To bring forth accurate key themes to describe a phenomena, Colaizzi devised seven steps of the analysis which aim to ensure vigour and trustworthiness of the research material.

To acquire a sense of each transcript, the first researcher (PH) will initially listen to the audio recording of each interview. This researcher (PH) will write down any thoughts and feelings that may arise while listening to the interviews in a reflective diary to assist with the bracketing process. It is important that bracketing is part of the analysis process to avoid any of the researcher’s preconceptions or assumptions [47]. The researcher will then transcribe all audio recorded interviews and subsequently identify and extract significant phases and statements that describe the participant’s experience. The transcripts will then be sent to other research team members (CB, AMH) to provide independent identification of significant themes and to review if the interpretive process of the data was clear in regard to the relationships, clusters and emergent themes. The theme clusters and emergent themes will then be integrated and presented as an exhaustive description of experiences that the participants have when accessing evidence based falls prevention recommendations. The resulting presentation of the phenomena will then be reduced by all three researchers to an essential structure [46] to explain the falls experience among older adults with ID. This final structure will also subsequently be integrated with the quantitative statistical findings in order to add value and explain the relationship between the nature of the falls and the experiences of participants.

## Discussion

There has been an increased attention and interest about facilitating “Ageing in Place” for people with disability, which operates on the principles of providing older adults with ID the choice of staying in their preferred residence and engaging within communities that are familiar to them [48]. Falls are known to frequently cause serious injuries such as fractures among older adults with ID [8, 9], which could lead to a negative impact on HRQoL [49]. It is important to understand the extent of the problem of falls in this population so as to provide targeted and effective services. Presently there are limited services that have been specifically designed or tested for addressing falls prevention among people with ID [22].

This study has been designed in accordance with recommendations for conducting falls research [16]. The study targets participants 35 years and older as it has been observed that people with ID start to develop age related changes from the mid to late third decade of their lives [5]. This study will also explore participants’ experiences when accessing falls prevention services after they experience a fall. This will assist to understand what barriers could be present when older adults with ID attempt to access falls prevention services and undertake relevant interventions.

Falls research guidelines recommend that falls data should be prospectively collected for 12 months [16]; however, the study is only collecting falls data for six months. Most of the participants are not likely to be able to complete the recordings independently and thus the tasks of recording falls will be mainly undertaken by paid and unpaid caregivers. The length of the observational period has been chosen as being less burdensome to participants and their caregivers, while still providing robust data about falls rates among older people living with ID. We have attempted to use strategies to increase the likelihood of falls data being collected reliably, such as building a rapport with the caregivers and increasing their awareness about the importance of falls prevention/management [14], but this may form a limitation if there are incomplete data. We are unable to collect data on falls that are unwitnessed and not subsequently reported to the caregiver by the person with ID. We are also likely to have incomplete data where participants are found on the floor and are unable to tell their caregiver or family what occurred. Data on fear of falls are not collected although recommended [50], as piloting demonstrated that it was difficult to seek this type of response directly and consistently from potential participants. Some pilot work has shown that this could possibly be obtained for some people with ID by proxy reporting, but given the reality that multiple caregivers are involved in different care environments (e.g. supported living, family home, community), proxy reporting may not be reliable [50].

The study is only advertised through one supporting organisation in WA that provides services to older adults with ID. The information of the study may not reach to others who do not have relationships/partnerships with the supporting organisation and therefore the sample may not be representative of the wider population of older adults with ID. However, this study will collect detailed information which could be used by other organisations with similar populations.

This study will provide robust data about the rate of falls and injurious falls among older adults with ID. It will also assist to identify how older adults with ID access health care after they experience a fall and barriers, if any, that they may encounter when seeking health care to reduce their risk of falls and falls related injuries. The results will also inform future development of guidelines and strategies for preventing falls among older adults with ID.

## Abbreviations

HRQol: Health related quality of life
ID: Intellectual Disability
WA: Western Australia
